# 129S1/SvImJ mice display impaired contextual fear extinction, enhanced fear incubation and deficit extinction consolidation phenotypes: rescue via pharmacological and non-pharmacological treatments

**DOI:** 10.1186/1471-2210-11-S2-A39

**Published:** 2011-09-05

**Authors:** Nigel Whittle, Markus Hauschild, Marguerite Camp, Andrew Holmes, Nicolas Singewald

**Affiliations:** 1 Department of Pharmacology and Toxicology, Institute of Pharmacy, and Center for Molecular Biosciences Innsbruck (CMBI), University of Innsbruck, 6020 Innsbruck, Austria; 2 Section of Behavioral Science and Genetics, Laboratory for Integrative Neuroscience, National Institute on Alcoholism and Alcohol Abuse, National Institutes of Health, Bethesda, MD 20852, USA

## Background

We recently revealed that 129S1/SvImJ (S1) mice exhibit deficits in engaging associative mechanisms to extinguish cued emotional responses. Here, we aimed to further characterise emotional responding in S1 and determine the effect of novel treatments on aberrant emotional responding.

## Methods and results

The present experiments firstly tested whether deficits in extinguishing emotional responses in S1 extends to contextual extinction. No reduction in emotional responses to the context was observed during an extinction training session which indicates generalised inability of S1 to engage associative fear extinction mechanisms. This was in contrast to mice of another 129 strain, 129S6/SvEvTac (S6), which showed contextual (and cued) extinction of emotional responding. Importantly, S1 and S6 showed identical responses on the flinch/jump test, which indicated that the results obtained were not an artefact of altered sensitivity to the unconditioned stimulus (US). Evidence that S1 mice can engage non-associative mechanisms to extinguish emotional responses was revealed as complete abolishment of contextual emotional expression was observed following US habituation (devaluing emotional responding to the US). We next investigated whether impaired cued emotional extinction persisted following also very “weak” conditioning paradigms. Results revealed that under these conditions, S1 mice displayed cued emotional (within) extinction when extinction training was performed using massed-CS presentations. This result indicates that the strength of the conditioning determines the ability of S1 to engage associative mechanisms to extinguish emotional responding. Using this paradigm it was possible to reveal impaired extinction consolidation in S1, as no between-session extinction was observed. Post extinction training application of D-cycloserine (an NMDA receptor agonist) and MS-275 (an HDAC inhibitor) rescued impaired extinction consolidation in S1. Finally, using “weak” conditioning, an enhanced sensitivity in S1 to incubate emotional responses was observed. During extinction training, temporally spaced CS presentations increased emotional responding in S1, but not the comparator strain C57BL6, revealing that S1 display an increased propensity over “normally” behaving mice to incubate emotional responses (via non-associative mechanisms) following weak trauma.

## Conclusions

Taken together, the present results suggest the utility of S1 to explore mechanisms underlying impaired contextual extinction, impaired extinction consolidation and enhanced emotional incubation.

